# Personal Goal-Related Mental Time Travel and Its Association With Resting-State Functional Connectivity in Individuals With High Schizotypal Traits

**DOI:** 10.1093/schbul/sbad183

**Published:** 2025-03-04

**Authors:** Jun-yan Ye, Xiao-jing Qin, Ji-fang Cui, Jia-li Liu, Hai-song Shi, Tian-xiao Yang, Ya Wang, Raymond C K Chan

**Affiliations:** Neuropsychology and Applied Cognitive Neuroscience Lab, CAS Key Laboratory of Mental Health, Institute of Psychology, Beijing, China; Department of Psychology, University of Chinese Academy of Sciences, Beijing, China; Neuropsychology and Applied Cognitive Neuroscience Lab, CAS Key Laboratory of Mental Health, Institute of Psychology, Beijing, China; Department of Psychology, University of Chinese Academy of Sciences, Beijing, China; Department of psychology, Guangxi Medical University, Nanning, China; Research Center for Information and Statistics, National Institute of Education Sciences, Beijing, China; Neuropsychology and Applied Cognitive Neuroscience Lab, CAS Key Laboratory of Mental Health, Institute of Psychology, Beijing, China; Department of Psychology, University of Chinese Academy of Sciences, Beijing, China; Mental Health Education Center, North China Electric Power University, Beijing, China; Neuropsychology and Applied Cognitive Neuroscience Lab, CAS Key Laboratory of Mental Health, Institute of Psychology, Beijing, China; Department of Psychology, University of Chinese Academy of Sciences, Beijing, China; Neuropsychology and Applied Cognitive Neuroscience Lab, CAS Key Laboratory of Mental Health, Institute of Psychology, Beijing, China; Department of Psychology, University of Chinese Academy of Sciences, Beijing, China; School of Psychology, Capital Normal University, Beijing, China; Neuropsychology and Applied Cognitive Neuroscience Lab, CAS Key Laboratory of Mental Health, Institute of Psychology, Beijing, China; Department of Psychology, University of Chinese Academy of Sciences, Beijing, China

**Keywords:** mental time travel, personal goal-advantage effect, neural, schizotypal

## Abstract

**Background and Hypothesis:**

Mental time travel (MTT) is a crucial ability for daily life. Personal goal-related MTT events has stronger phenomenological characteristics than personal goal-unrelated ones, ie, the “personal goal-advantage effect”. However, it remains unclear whether this effect is impacted in individuals with high schizotypal traits (HST) and the neural correlates of this effect have yet to be elucidated. The present study aimed to fill these knowledge gaps. We hypothesized that HST would show a reduced “personal goal-advantage effect” in MTT and would exhibit altered relationships with resting-state functional connectivity.

**Study Design:**

In Study 1, 37 HST and 40 individuals with low schizotypal traits (LST) were recruited. Participants generated MTT events with personal goal-related and personal goal-unrelated cues. In Study 2, 39 HST and 38 LST were recruited, they completed the same behavioral task and resting-state functional magnetic resonance imaging (fMRI) scanning.

**Study Results:**

Both Study 1 and Study 2 revealed that HST exhibited reduced “personal goal-advantage effect” on MTT specificity. Moreover, Study 2 showed that compared with LST, HST exhibited altered association between the “personal goal-advantage effect” and functional connectivity (ie, between the right precuneus and the left postcentral gyrus and “personal goal-advantage effect” on emotional valence, between the left hippocampus and the right temporal fusiform gyrus and “personal goal-advantage effect” on emotional intensity).

**Conclusions:**

These findings suggest that HST exhibit a reduced “personal goal-advantage effect” in MTT specificity and altered neural correlates related to this effect. The “personal goal-advantage effect” may be a potential target for intervention in HST.

## Introduction

Mental time travel (MTT) refers to the ability to re-experience past events (autobiographical memory, AM) and to pre-experience future events (episodic future thinking, EFT) through mental simulation.^1,2^ MTT plays a crucial role in various functions such as planning, decision making, emotion regulation, and problem solving.^3–5^ The literature suggests that patients with schizophrenia exhibit significant impairments in MTT.^6,7^ Recent findings further suggest that people with subclinical features such as high levels of schizotypal traits (HST) also exhibit an attenuated impairment in cognitive and emotional processing.^8–10^ However, whether people with HST are impaired in MTT remains unclear. For example, some previous studies have shown that people with HST reported more details and stronger sense of experience during MTT,^11^ whereas other studies reported less vividness, weaker sense of experience, fewer details, and lower positive emotions in HST individuals’ MTT.^12–18^ In addition, some previous studies did not find any significant difference in MTT between people with high vs low levels of schizotypal traits (HST vs LST).^18,19^ Further studies are needed to clarify the association between schizotypal traits and MTT.

MTT relies on the autobiographical knowledge,^20–22^ which is believed to have a hierarchical structure, such that general themes are stored at the higher level, general past events at the medium level, and specific past episodes at the lower level.^20,23^ In the MTT processing, individuals would access their autobiographical knowledge store, and retrieve past events in abstract forms, such as general themes. This abstract information would then be modified, using information which bear personal significance or align with personal goals. The modified abstract events would subsequently integrate with semantic memory and contextual framework to form general events. Based on the general events, individuals can simulate scenes and perceptual experiences, and generate “meaningful and specific” events. Recent studies indicated that EFT and AM undergo similar MTT processing.^20,23,24^ Individuals can organize past events and future events cohesively and map these events onto their personal timeline with the autobiographical knowledge structure.^21,22^

Personal goals are important to direct an individual to strive, and are highly self-relevant.^25^ MTT events related to personal goals are heavily processed in the autobiographical knowledge structure, and have stronger phenomenological characteristics. Previous studies showed that personal goal-related MTT events were more specific^26^ and vivid,^27^ with stronger sense of experience^28,29^ and positive emotions,^27,30^ than personal goal-unrelated MTT events. This phenomenon has been termed “personal goal-advantage effect” in MTT, this effect facilitates individuals to construct a coherent sense of self,^29^ and promotes life satisfaction.^27,30,31^ Despite that “personal goal-advantage effect” is crucial in MTT, it has not been well studied in people with HST. Personal goals can be further divided into approach goals and avoidance goals,^32,33^ depending on the motivation source and outcome orientation. One previous study found that patients with major depressive disorder (MDD) generated less specific approach and avoidance goals, and fewer specific explanations for approach goals compared to healthy controls.^34^ The approach-avoidance differentiation of personal goals in people with HST has not yet been studied.

The process of MTT involves the “core network,”^35^ which includes the prefrontal cortex, hippocampus, parahippocampal gyrus, precuneus, cingulate gyrus, and temporoparietal junction area.^35–38^ A meta-analysis showed that personal goal processing and EFT co-activated several brain regions of the core network, including the medial prefrontal cortex, posterior cingulate gyrus, left middle temporal gyrus, and right parahippocampal gyrus.^39^ The medial prefrontal lobe is involved in evaluating the relevance of simulated events to personal goals and adding more phenomenal characteristics to MTT events. The posterior cingulate gyrus is involved in allocating attentional resources towards goal-related stimuli and constructing and evaluating MTT events.^40^

Limited studies on the neural mechanisms underlying MTT in people with HST have been conducted. In a resting-state fMRI study, individuals with social anhedonia (ie, negative schizotypal traits) showed weakened functional connectivity between the posterior cingulate cortex and the right superior temporal gyrus, which was associated with reduced details in EFT.^41^ In another MRI study in individuals with social anhedonia, the functional connectivity between the amygdala and posterior cingulate cortex was negatively correlated with anticipated emotional valence in EFT, whereas the functional connectivity between the hippocampus and parahippocampal gyrus was positively associated with anticipated emotional valence.^17^ In task-based fMRI studies, individuals with social anhedonia were found to have reduced activation in the right caudate and right precuneus, but stronger functional connectivity between the caudate and lingual gyrus during positive EFT,^18^ and have higher activation in bilateral caudate and the thalamus during positive EFT.^16^ Moreover, they exhibited lower activation in bilateral anterior cingulate cortex and the right medial superior frontal gyrus.^15^ However, none of the aforementioned studies investigated the “personal goal-advantage effect” on MTT in individuals with HST.

Taken together, the relevance of simulated events to personal goals can enhance MTT phenomenal characteristics. However, it remains unclear whether such “personal goal-advantage effect” would be altered in individuals with HST. We aimed to fill these knowledge gaps through two inter-related studies. Study 1 aimed to examine the “personal goal-advantage effect” in individuals with HST; whereas Study 2 sought to replicate the Study 1 results, and further investigate the neural correlates of “personal goal-advantage effect” using resting-state MRI. Based on previous findings,^12–18^ we hypothesized that the “personal goal-advantage effect” would be reduced in individuals with HST. We also hypothesized that the associations between this effect and resting-state functional connectivity would differ between individuals with LST and HST.

## Study 1

### Methods

#### Participants.

The Schizotypal Personality Questionnaire (SPQ)^42,43^ was used to identify subjects with high schizotypal traits (HST) and low schizotypal traits (LST). Using the SPQ cut-off scores of ≥42 for HST and ≤26 for LST, we recruited 37 HST and 40 LST participants. The inclusion and exclusion criteria are shown in Supplementary materials (Study 1 Participants section). This study was approved by the Ethics Committee of the Institute of Psychology, Chinese Academy of Sciences (No. H20039). Written informed consent was obtained from participants.

### Measures

#### Personal Goal MTT Task.

This task was adapted from the Personal Goal-Directed Simulation Task^27^ and the Personal Goal Imagination Task,^31^ and comprised two phases: (1) personal goal generation phase and (2) MTT narrative phase, which were conducted one day apart. In the first phase, participants were instructed to generate 12 personal goals (6 approach goals, 6 avoidance goals), and rate their phenomenal characteristics (ie, importance, possibility, centrality, difficulty, sense of happiness, sense of sadness) on a Likert scale (0 = not at all, 100 = very much). Then participants chose 6 personal goal-unrelated cues from a predefined list. In the second phase, participants were instructed to recall and simulate personal goal-related and personal goal-unrelated MTT events, after viewing the cues given in the first phase. They then gave a narrative description of each of the MTT events, and ratings of vividness (1 = not vivid at all, 10 = very vivid), sense of experience (1 = not strong at all, 10 = very strong), emotional valence (1 = very negative, 5 = very positive), emotional intensity (1 = not strong at all, 5 = very strong), and difficulty in generating the MTT events (1 = not at all, 5 = very difficult). All descriptions were audio-recorded and later transcribed into text and the events were then rated according to the manual.^12,44,45^ The proportion of specific events (ie, lasting <24 h and including contextual information of time, place, perceptual details, etc.) was the variable of interest for further analysis.

**Fig. 1.**
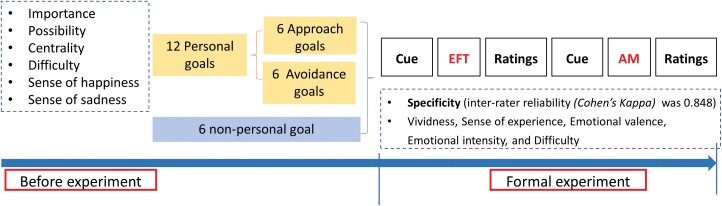
Procedure of personal goal mental time travel task.

Before the formal experiment, participants completed a practice trial to ensure that they understood the task requirements. Figure 1 shows the procedures of the paradigm. The details of the materials are presented in Supplementary materials (Materials and Procedure sections).

#### Other Cognitive Measures.

IQ was estimated from the short-form of the Chinese version of the Wechsler Adult Intelligence Scale-Revised,^46^ including Information, Arithmetic, Similarity, and Digit Span. We also administered the verbal fluency test and estimated the number of correct response.^47^

### Data Analysis

Two-sample *t*-tests and Chi-square tests were used to compare demographic information and goal characteristics between the HST and the LST groups. We used 2 (Group: HST, LST) × 2 (Goal type: Personal goals, Personal goal-unrelated) × 2 (Time orientation: AM, EFT) repeated measure ANOVAs, with MTT indices as the dependent variables. If the main effect of Goal type or interactions between Goal type and Group were significant, we further examine the differential effect of approach—avoidance goals. The significance level was set at *P* < .05. All analyses were completed using SPSS 22.0. Given our main objective was to investigate the “personal goal advantage effect” in HST vs LST individuals, we focused on the Group and Goal type main effects and the interaction effects of these two variables. The remaining results are shown in Supplementary materials (Study 1 Results section).

## Results

The HST and LST groups were comparable in terms of age (Mean(*SD*)_HST_ = 21.16 (2.78), Mean(*SD*)_LST_ = 21.18 (2.51), *t*_(75)_ = −0.02, *P* = .983), education (Mean(*SD*)_HST_ = 14.89 (1.73), Mean(*SD*)_LST_ = 15.08 (2.20), *t*_(75)_ = −0.40, *P* = .687), estimated IQ (Mean(*SD*)_HST_ = 118.00 (14.00), Mean(*SD*)_LST_ = 121.00 (12.93), *t*_(75)_ = −0.98, *P* = .331), verbal fluency (Mean(*SD*)_HST_ = 24.59 (4.47), Mean(*SD*)_LST_ = 24.82 (2.97), *t*_(75)_ = 0.26, *P* = .799), and characteristics of goals (see Supplementary tables S2 and S3).

We found a significant interaction between Group and Goal type on MTT specificity (*F*_(1,75)_ = 5.88, *P* = .018, η_p_^2^ = 0.07). Simple effect analysis revealed that the LST group generated more specific events for personal goal cues than personal goal-unrelated cues (*t*_(75)_ = 4.59, *P* < .001, Cohen’s *d* = 0.61), whereas the HST group showed similar MTT in response to goal-related and goal-unrelated cues (*t*_(75)_ = 1.06, *P* = .295, Cohen’s *d* = 0.15). These results suggested a weaken “personal goal-advantage effect” on specificity in the HST group.

The Group main effect was significant on emotional valence (*F*_(1, 75)_ = 6.73, *P* = .011, η_p_^2^ = 0.08). The HST group generated events with less positive emotion than the LST group. Moreover, the Goal type main effect was significant for specificity (*F*_(1,75)_ = 15.56, *P* < .001, η_p_^2^ = 0.17), vividness (*F*_(1,75)_ = 27.14, *P* < .001, η_p_^2^ = 0.27), sense of experience (*F*_(1,75)_ = 49.78, *P* < .001, η_p_^2^ = 0.40), emotional intensity (*F*_(1,75)_ = 48.76, *P* < .001, η_p_^2^ = 0.39), and difficulty in MMT (*F*_(1,75)_ = 6.48, *P* = .013, η_p_^2^ = 0.08). Personal goal-related events showed higher specificity, vividness, sense of experience, emotional intensity, and lower difficulty in MTT, compared to personal goal-unrelated events (see table 1). The results regarding the approach-avoidance differentiation are presented in Supplementary table S4.

**Table 1.** The Phenomenological Characteristics of Personal Goal-related and Personal Goal-unrelated MTT in Study 1

	HST				LST				Group		Goal type		Group × goal type	
	PG		NPG		PG		NPG		F	η_p_^2^	F	η_p_^2^	F	η_p_^2^
	Mean	SD	Mean	SD	Mean	SD	Mean	SD	*df* = _(1,75)_		*df* = _(1,75)_		*df* = _(1,75)_	
Specificity^#^ (0–1)														
AM	0.66	0.22	0.64	0.26	0.75	0.20	0.68	0.25	3.75	0.05	15.56***	0.17	5.88*	0.07
EFT	0.63	0.20	0.58	0.21	0.78	0.17	0.58	0.29						
Vividness (1–10)														
AM	7.32	1.16	7.10	1.05	7.61	1.04	7.32	1.14	1.89	0.03	27.14***	0.27	0.01	<0.01
EFT	6.72	1.27	6.01	1.20	7.07	1.18	6.40	1.21						
Sense of experience (1–10)														
AM	7.41	1.44	7.03	1.27	7.69	0.93	7.17	1.06	10.48	0.02	49.78***	0.40	0.02	<0.01
EFT	6.27	1.26	5.29	1.40	6.57	1.25	5.67	1.28						
Valence (1–5)														
AM	2.95	0.52	3.17	0.33	3.07	0.37	3.21	0.35	6.73*	0.08	1.29	0.02	0.42	0.01
EFT	3.25	0.55	3.21	0.42	3.54	0.45	3.45	0.47						
Intensity (1–5)														
AM	3.15	0.55	2.87	0.52	3.21	0.52	2.95	0.58	0.93	0.01	48.76***	0.39	0.89	0.01
EFT	3.29	0.59	2.71	0.48	3.34	0.54	2.95	0.68						
Difficulty (1–5)														
AM	1.64	0.50	1.73	0.54	1.52	0.43	1.55	0.49	2.85	0.04	6.48*	0.08	0.19	<0.01
EFT	2.00	0.56	2.21	0.59	1.82	0.54	2.00	0.61						

*Note*: AM, autobiographical memory; EFT, episodic future thinking; HST, high schizotypal traits, LST, low schizotypal traits; NPG, personal goal-unrelated; PG, personal goal-related.

*P < .05; **P < .01; ***P < .001; Group = high schizotypal traits vs low schizotypal traits; MTT = autobiographical memory vs episodic future thinking; Goal type = Personal goal-related vs personal goal-unrelated; Group × goal type = group and goal type interaction.

^#^Proportion of specific events.

## Discussion

Consistent with our hypothesis, we found weaken “personal goal-advantage effect” on MTT for specificity in the HST group. The LST group generated more specific events for personal goal-related cues than personal goal-unrelated cues. Our findings that the HST group generated events with less positive emotions were consistent with previous research.^41^ The “personal goal-advantage effect” of MTT was observed in this study, consistent with previous findings.^26,28–30^

## Study 2

### Methods

#### Participants.

We recruited 39 HST and 38 LST participants using the eligibility criteria as detailed in Supplementary materials (Study 2 Participants section). Among Study 2 sample, 8 HST participants and 14 LST participants were originated from Study 1 sample. This study was approved by the Ethics Committee of the Institute of Psychology, the Chinese Academy of Sciences (No. H20039). Informed consent was obtained from all participants.

### Measures

#### MTT Task and Other Cognitive Measures.

Participants first completed the behavioral task, then they underwent imaging scanning. We used the same personal goal mental time travel task, and cognitive measures as in Study 1.

#### MRI Data Acquisition.

Imaging data were acquired using a General Electric Discovery MR 750 scanner at the Institute of Psychology, Chinese Academy of Sciences. Resting-state fMRI data were acquired using T2-weighted echo planar imaging sequence, with the following parameters: 240 volumes, TR = 2000 ms, TE = 30 ms, flip angle = 90°, slice thickness = 3.5 mm, slice number = 37, matrix size = 64 × 64, FOV = 220 mm, and voxel size = 3.4 mm × 3.4 mm × 3.5 mm. The total scanning time for resting-state fMRI was 8 min. Moreover, structural MRI data were acquired using T1-weighted Magnetization Prepared Rapid Gradient Echo sequence, with the following parameters: TR = 6.65 ms, TE = min full, TI = 450 ms, FOV = 256 mm, flip angle = 12°, number of slices = 192, slice thickness = 1 mm, matrix = 256 × 256, voxel size = 1 mm × 1 mm × 1 mm. During scanning, spongy pads were used to stabilize participants’ heads.

### Data Analysis

#### Image Data Analysis.

Preprocessing for resting-state fMRI data was conducted with the DPABI^48^ and the CONN (The Conn Functional Connectivity Toolbox)^49^ toolboxes based on MATLAB R2014a (MathWorks Inc., Natick, MA, USA). Detailed information regarding imaging data preprocessing is shown in Supplementary materials (Study 2 Preprocessing and denoising of MRI data section).

We selected eight regions of interest (ROIs) to represent the core network related to MTT^50^ and two other ROIs to represent personal goal processing^39^ with a radius of 6 mm (see Supplementary table S1 for coordinates). The mean time series for each ROI was calculated in each participant, and then Pearson’s correlations were used to calculate the associations between the mean time series for each ROI and the rest of the whole-brain voxels for each participant. Fisher’s *r*-to-*z* transformation was used to obtain Fisher’s *Z*-value maps for each participant for all ROIs for subsequent regression analyses.

To examine the differences in the pattern of functional connectivity related to the “personal goal-advantage effect” on MTT between the HST and the LST groups, we used group-level multiple regression model in the CONN toolbox. We included Fisher’s *Z*-value map for each ROI in the regression model, with Group and the “personal goal-advantage effect” for each MTT phenomenological characteristic score as the regressors, and age, gender, and the FD-power mean as the covariates to build the regression model. The significance level was set as *P* < .005 (uncorrected) at the voxel level,^51,52^ and cluster-level Family-wise error (FWE) corrected *P*_FWE_ < .005 at the cluster-level (where 0.005 = 0.05/10 indicates additional correction for 10 ROIs) with a cluster size of over 20 voxels.^15,52^ BrainNet Viewer was used for visualization.

## Results

### Demographic and Behavioral Results

The HST and LST groups showed comparable age (Mean(SD)_HST_ = 20.67 (2.13), Mean(SD)_LST_ = 20.37 (1.44), *t*_(75)_ = 0.72, *P* = .475), education (Mean(SD)_HST_ = 14.46 (1.67), Mean(SD)_LST_ = 14.55 (1.20), *t*_(75)_ = −0.27, *P* = .785), estimated IQ (Mean(SD)_HST_ = 122.64 (10.05), Mean(SD)_LST_ = 122.34 (9.62), *t*_(75)_ = 0.13, *P* = .894), verbal fluency (Mean(SD)_HST_ = 24.56 (3.28), Mean(SD)_LST_ = 25.53 (4.67), *t*_(75)_ = −1.05, *P* = .297), and characteristics of goals (see Supplementary tables S5 and S6).

The interaction between Group and Goal type was significant for specificity (*F*_(1,75)_ = 6.20, *P* = .015, η_p_^2^ = 0.08) (see table 2). Simple effect analysis showed that the HST group generated fewer specific personal goal-related events than the LST group (*t*_(150)_ = −4.41, *P* < .001, Cohen’s *d* = −0.84), but the two groups generated similar number of specific personal goal-unrelated events (*t*_(150)_ = −1.66, *P* = .102, Cohen’s *d* = −0.32). These results suggested a weakened “personal goal-advantage effect” for specificity in the HST group.

**Table 2.** The Phenomenological Characteristics of Personal Goal-related and Personal Goal-unrelated MTT in Study 2

	HST				LST				Group		Goal type		Group × goal type	
	PG		NPG		PG		NPG		*F*	η_p_^2^	F	η_p_^2^	F	η_p_^2^
	Mean	SD	Mean	SD	Mean	SD	Mean	SD	*df* = _(1,75)_		*df* = _(1,75)_		*df* = _(1,75)_	
Specificity^#^ (0–1)														
AM	0.56	0.24	0.64	0.26	0.71	0.20	0.70	0.22	13.24***	0.15	1.60	0.02	6.20*	0.08
EFT	0.50	0.22	0.60	0.28	0.75	0.23	0.70	0.25						
Vividness (1–10)														
AM	7.16	1.36	7.01	1.42	7.64	1.10	7.36	1.26	3.81	0.05	14.74***	0.16	0.29	<0.01
EFT	6.34	1.22	5.82	1.41	6.95	1.11	6.33	1.27						
Sense of experience (1–10)														
AM	7.12	1.42	6.88	1.53	7.46	1.19	7.00	1.32	0.94	0.01	24.09***	0.24	0.54	0.01
EFT	5.95	1.26	5.33	1.78	6.30	1.28	5.60	1.45						
Valence (1–5)														
AM	2.97	0.38	3.16	0.45	3.05	0.40	3.32	0.30	3.54	0.05	2.45	0.03	1.73	0.02
EFT	3.44	0.48	3.28	0.42	3.49	0.52	3.47	0.46						
Intensity (1–5)														
AM	3.09	0.47	2.88	0.55	3.14	0.63	2.82	0.78	0.03	<0.01	41.83***	0.36	1.56	0.02
EFT	3.22	0.57	2.83	0.60	3.26	0.58	2.71	0.86						
Difficulty (1–5)														
AM	1.61	0.53	1.64	0.46	1.48	0.41	1.54	0.55	1.57	0.02	6.02*	0.07	0.04	<0.01
EFT	1.95	0.52	2.20	0.73	1.84	0.50	2.01	0.68						

*Note*: AM, autobiographical memory; EFT, episodic future thinking; HST, high schizotypal traits, LST, low schizotypal traits; NPG, personal goal-unrelated; PG, personal goals.

*P < .05; **P < .01; ***P < .001; Group = high schizotypal traits vs low schizotypal traits; MTT = Autobiographical memory vs Episodic future thinking; Goal type = Personal goals vs Personal goal-unrelated; Group × Goal type = Group and Goal type interaction.

^#^Proportion of specific events.

Regarding the Group main effect, the HST group generated fewer specific events (*F*_(1, 150)_ = 13.24, *P* < .001, η_p_^2^ = 0.15) than the LST group (see table 2). The Goal type main effect was significant for vividness (*F*_(1,75)_ = 17.74, *P* < .001, η_p_^2^ = 0.16), sense of experience (*F*_(1,75)_ = 24.09, *P* < .001, η_p_^2^ = 0.24), emotional intensity (*F*_(1,75)_ = 41.83, *P* < .001, η_p_^2^ = 0.36), and difficulty in MTT (*F*_(1,75)_ = 6.02, *P* = .016, η_p_^2^ = 0.07). Personal goal-related events showed higher vividness, sense of experience, emotional intensity, and lower generation difficulty compared to personal goal-unrelated events (see table 2). The results regarding the approach-avoidance differentiation are presented in Supplementary materials (table s7).

### Associations Between Resting-State Functional Connectivity and the “personal goal-advantage effect”

**Fig. 2.**
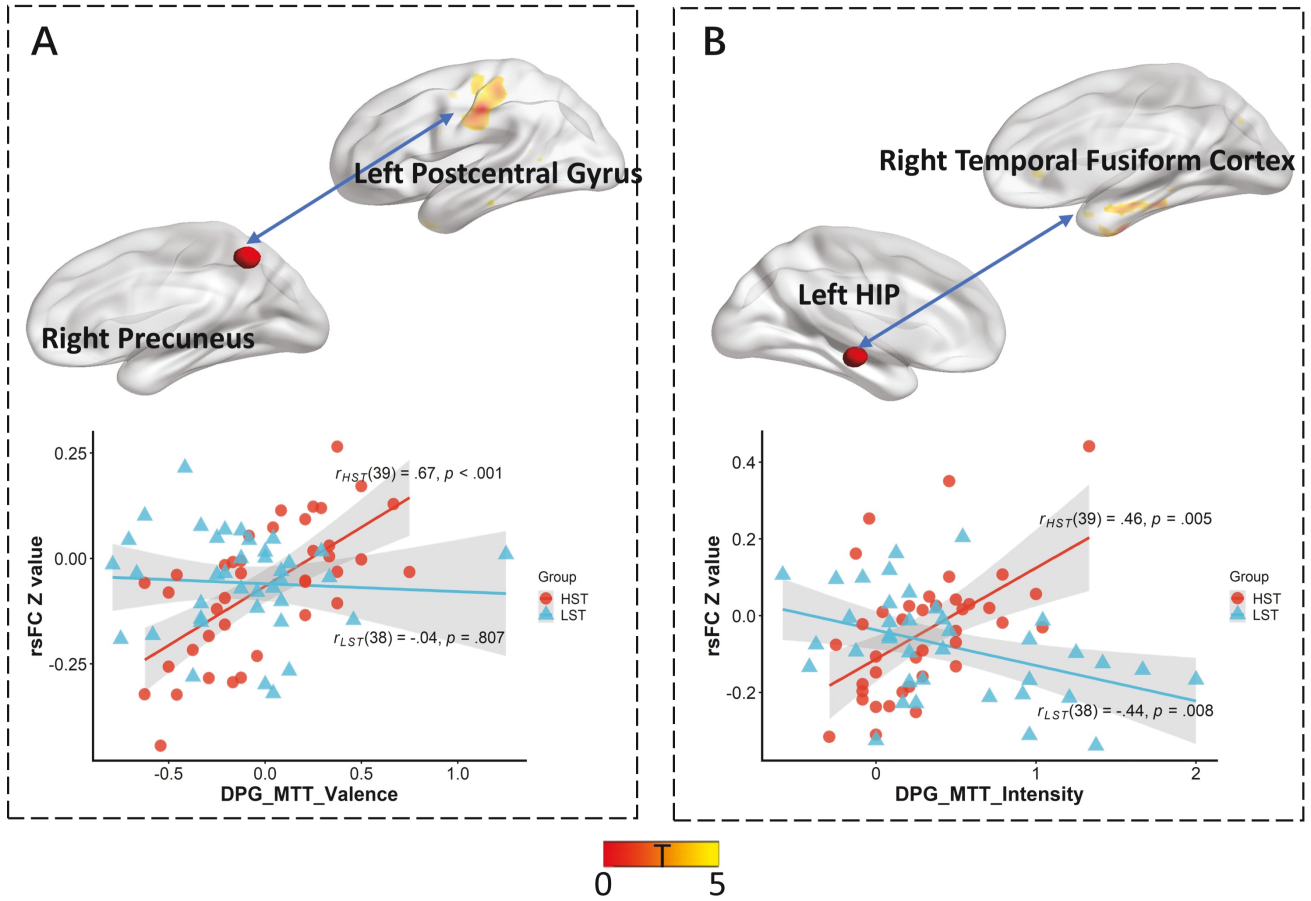
Significant group difference of the relationship between personal goal-advantage effect on MTT and functional connectivity. DPG_MTT_Valence = The "personal goal-advantage effect" on emotional valence; DPG_MTT_Intensity =The "personal goal-advantage effect" on emotional intensity; rsFC = resting-state functional connectivity; HIP = hippocampus. (**A**) Non-significant correlation between the functional connectivity of the right precuneus and the left postcentral gyrus with personal goal-advantage effect on emotional valence in LST, but this correlation was positive in HST. (**B**) The negative correlation between the functional connectivity of the left hippocampus and the right temporal fusiform gyrus with personal goal-advantage effect on emotional intensity in LST, but this correlation was positive in HST. Threshold: cluster-level FWE adjusted *P* < .05, cluster size >20 (voxel-level uncorrected *P* < .005), multiple comparison correction *P*_FWEcorrection_ = .005.

The difference between the personal goal-related and personal goal-unrelated MTT phenomenological characteristic scores was calculated to represent the “personal goal-advantage effect.” The associations between resting-state functional connectivity and the “personal goal-advantage effect” in MTT were calculated in the HST and the LST groups. The two groups showed significant difference in the two correlations after combined multiple comparison corrections (*P*_FWE_ < .005) (see table 3 and figure 2).

**Table 3.** The Group Difference of the Relationship Between the Personal Goal-ADVANTAGE Effect in MTT and Functional Connectivity

Personal goal-advantage effect	ROIs	Regions	*T*	MNI coordinates			Cluster size	*P*_FWE_	HST (*r*, *P*)	LST (*r*, *P*)
				*X*	*Y*	*Z*				
Emotional valence	Right precuneus	Left postcentral gyrus	−5.20	−54	−21	42	70	.001	.67 <.001	−.04 .807
Emotional intensity	Left hippocampus	Right temporal fusiform cortex	−7.47	42	−6	−39	76	.001	.65 <.001	−.58 <.001

*Note*: HST, high schizotypal traits; LST, low schizotypal traits; MTT, mental time travel; threshold: cluster-level FWE adjusted *P* < .005, cluster size >20 (voxel-level uncorrected *P* < .005).

As shown in figure 2A, the correlation between the functional connectivity (*Z*-values) of the right precuneus (Precuneus_R) and the left postcentral gyrus and the “personal goal-advantage effect” on emotional valence showed significant group difference (*t* = −5.20, *P*_FWE_ = .001). There was no significant association in the LST group (*r* = −.04, *P* = .807), but there was a positive association in the HST group (*r* = .67, *P* < .001).

As shown in figure 2B, the correlation between the functional connectivity (*Z*-values) of the left hippocampus (HIP_L) and the right temporal fusiform gyrus and the “personal goal-advantage effect” on emotional intensity showed significant group difference (*t* = −7.47, *P*_FWE_ = .001). The association was negative in the LST group (*r* = −.58, *P* < .001), and positive in the HST group (*r* = .65, *P* < .001). The results that uncorrected for number of ROIs are presented in Supplementary figure S1 and table S8.

## Discussion

Study 2 investigated the relationship between functional connectivity and the “personal goal-advantage effect” in HST individuals. Study 2 results were consistent with Study1 results, that the “personal goal-advantage effect” on MTT specificity had diminished in HST individuals. Our findings also support the existence for “personal goal-advantage effect” on MTT for ratings of vividness, sense of experience, emotional intensity, and difficulty in MTT.

More importantly, HST individuals showed an altered pattern of relationship between the “personal goal-advantage effect” and resting-state functional connectivity. Specifically, we found significant correlations of (1) the connectivity between the right precuneus and the left postcentral gyrus with the “personal goal-advantage effect” on emotional valence, and (2) the connectivity between the left hippocampus and the right temporal fusiform gyrus with the “personal goal-advantage effect” on emotional intensity.

## General Discussion

Taken together, our findings supported the “personal goal-advantage effect” on MTT, and suggested putative neural correlates of such phenomenon in HST individuals. Specifically, both Study 1 and Study 2 findings indicated that people with HST had diminished “personal goal-advantage effect” on MTT for specificity. Moreover, Study 2 findings suggested that the association of functional connectivity between the right precuneus and the left postcentral gyrus with the “personal goal-advantage effect” on emotional valence was altered in HST individuals. Moreover, the association of functional connectivity between the left hippocampus and the right temporal fusiform gyrus with the “personal goal-advantage effect” on emotional intensity was altered in HST individuals.

The HST and the LST groups differed in the “personal goal-advantage effect” on MTT for specificity, which may be attributable to personal goal-related events. Autobiographical events are stored in the autobiographical knowledge structure which is organized hierarchically.^20^ Personal goals link life events together.^29^ To simulate personal goal-related MTT events, the generation of details depends on both the number of details available and the interrelatedness or organization of the elements in the autobiographical knowledge structure.^53^ Studies have shown that HST individuals experienced reduced positive emotion during AM^14^ and EFT.^12,17,18,41^ It is plausible that impaired emotional processing may render HST individuals and schizophrenia patients having little pleasure while they simulate or retrieving past events, and thus having less motivation to engage goal-directed behaviors.^54–56^ Therefore, HST individuals may experience fewer personal goal-related events in their daily lives, and maintain less detailed information necessary for generating personal goal-related specific events.^12^ Furthermore, reduced goal-relevant experiences and expectations for goals may pose difficulty to HST individuals in organizing relevant details into coherent events.^17,57^ Considering the types of goals, we found that goal-related events were more vivid, positive, and emotionally intensive than goal-unrelated events, consistent with prior findings.^58^ More studies are needed to clarify the underlying mechanisms of the altered “personal goal-advantage effect” in people with HST.

People with HST also showed altered patterns of functional connectivity associated with the “personal goal-advantage effect.” This finding may reflect early changes and compensatory mechanisms in the brain networks. In this study, we found a positive correlation of the functional connectivity between the right precuneus and the left postcentral gyrus with the “personal goal-advantage effect” on emotional valence in HST participants rather than LST participants. The precuneus is involved in processing visual-spatial details and emotional information during MTT.^59,60^ Activation of the precuneus was associated with social goal processing.^61^ In addition, compared with LST counterparts, HST individuals showed reduced activation of the precuneus during positive future thinking.^18^ The postcentral gyrus is involved in sensory information processing and emotion regulation.^62,63^ The functional connectivity between the precuneus and the postcentral gyrus may reflect an exchange of sensory and emotional information between brain regions. In HST participants, we found a positive correlation of the functional connectivity between the precuneus and the postcentral gyrus with the “personal goal-advantage effect” on emotional valence, and this result may indicate possible compensatory mechanisms to maintain the “personal goal-advantage effect” on emotional valence.

In HST participants, we found a positive correlation of the functional connectivity between the left hippocampus and the right temporal fusiform gyrus with the “personal goal-advantage effect” on emotional intensity. By contrast, such correlation was negative in direction in LST participants. The hippocampus is involved in the storage, retrieval, and construction of scene details in MTT,^23,64^ and is responsible to increase the vividness and sense of experience in MTT.^65^ The fusiform gyrus and hippocampus are associated with richness of details.^41^ The altered functional connectivity-related pattern may represent possible compensatory mechanisms in HST participants. Previous research revealed that the functional connectivity between the fusiform gyrus and the postcentral gyrus was related to the richness of details.^41^ Our study unveiled the functional connectivity related to the fusiform gyrus, suggesting that the fusiform gyrus is an important brain region for visual and imagery processing in MTT.^50,66,67^ Higher richness of the details and sense of experience may elicit higher emotional intensity during MTT.^68^ There was no significant group difference on emotional intensity in the MTT task. This may suggest that HST individuals need stronger functional connectivity to maintain normal “personal goal-advantage effect” in emotional intensity in MTT, ie, the hyper-functional connectivity is a compensatory effect.

Similar degrees of AM and EFT impairments have been reported in patients with schizophrenia, major depressive disorder (MDD), bipolar disorders, and post-traumatic stress disorder (PTSD),^7,69,70^ although MDD patients were found to be particularly impaired in positive EFT.^7,71^ Furthermore, similar findings were found in subclinical individuals with schizotypal traits, autistic traits, and depressive symptoms in EFT.^16^ HST individuals and people with autistic traits also showed abnormal activation patterns in the core network during EFT.^16^ Therefore, there may be similar behavioral manifestations and neural mechanisms and also some unique patterns on the “personal goal-advantage effect” among different clinical and subclinical populations. Further studies are needed to examine this issue.

Studies on temporal processing reported that schizophrenia patients showed higher degrees of temporal imprecision between different trials such as lower similarity of single trial time courses in an event-related potential study.^72^ Another study provided evidence for basic temporal processing impairments in schizophrenia patients on different timescales (longer autocorrelation window and shorter intertrial phase coherence in EEG).^73^ These temporal processing difficulties might be related to MTT impairment in schizophrenia. However, further studies are needed to examine their associations directly.

Some limitations of this study should be borne in mind. Firstly, the HST population is heterogeneous,^74,75^ but we only compared participants who showed extreme ratings on the total score of the SPQ. Secondly, diminished “personal goal-advantage effect” on specificity was found in the HST group, while no group difference was found on the association between the “personal goal-advantage effect” for specificity and functional connectivity, further studies are needed to confirm the current results. Thirdly, the length of resting-state fMRI was relatively short, future studies should scan for a longer time to make the results more robust. Fourthly, in the functional connectivity analysis, we used a relatively lenient threshold, although this threshold has been adopted in previous studies,^15,51,52^ there is risk for false positive results. However, given that our study is preliminary and the participants were non-clinical, we think our results are valuable and require further validation. In addition, future studies could examine the potential changes in neural mechanisms in HST individuals using task-based fMRI. Finally, this is a preliminary study and further studies are needed in other groups along the schizophrenia spectrum, including ultra-high-risk individuals, first-episodic and chronic patients to track the trajectory along the spectrum.

Notwithstanding these limitations, our findings have implications. Personal goals facilitate the extraction and integration of situational details^76^ and induce more positive emotions,^27,28,30^ therefore, cognitive interventions targeting MTT based on personal goals may not only increase an individual’s willingness to pursue personal goals, but also enhance general MTT ability. In addition, the neuroimaging results can help select target areas for neuromodulation intervention to improve MTT.

## Conclusion

People with HST have a diminished “personal goal-advantage effect” on specificity and altered relationship patterns between the “personal goal-advantage effect” on MTT and functional connectivity.

## Supplementary Material

Supplementary material is available at https://academic.oup.com/schizophreniabulletin/.

sbad183_suppl_Supplementary_Tables_S1-S8_Figures_S1
