# Supplementary material for: Personal Goal-Related Mental Time Travel and Its Association With Resting-State Functional Connectivity in Individuals With High Schizotypal Traits
Source: Schizophr Bull. 2025 Mar 4;51(Suppl 2):S194–204. doi: 10.1093/schbul/sbad183 (PMC11879585; doi:10.1093/schbul/sbad183)
Supplement: sbad183_suppl_Supplementary_Tables_S1-S8_Figures_S1 [file sbad183_suppl_supplementary_tables_s1-s8_figures_s1.docx]

**Supplementary Materials**

**Additional method**

**Study 1 Participants**

A total of 426 college or graduate students completed the online schizotypal personality questionnaire (SPQ) (Chen, Hsiao, & Lin, 1997; Raine, 1991). The SPQ is a self-report questionnaire used to screen individuals with high and low schizotypal traits (HST, LST). It includes 74 “yes” (scored 1) or “no” (scored 0) items. According to the manual, participants who scored within the top 10% (SPQ score ≥ 42 in this study) were considered as HST, while those who scored below the mean (SPQ score ≤ 26 in this study) were considered as LST.

The additional inclusion criteria for both HST and LST were as follows: 1) without current or history of psychiatric diseases (based on the Mini International Neuropsychiatric Interview (MINI)) (Sheehan et al., 1998; Si et al., 2009); 2) no history of neurological diseases, drug or alcohol dependence; 3) aged between 18~26; 4) right-handed and with clear language expression. For the LST group, participants also need to have no family history of psychiatric diseases.

Sample size was calculated with G*Power v3.1.9.7 (Faul, Erdfelder, Lang, & Buchner, 2007), the statistical power was set as 0.8, α was 0.05, and medium effect size was Cohen's *d* = 0.25. Result showed that at least 28 participants were required in each group.

**Study 2 Participants**

A total of 991 college or graduate students completed the online schizotypal personality questionnaire (SPQ). Following the same criteria as Study 1, SPQ score above 42 were considered as HST, while those who scored below 26 were considered as LST.

In addition to inclusion criteria of Study 1, participants in both groups were required to have no fMRI contraindications. A total of 39 individuals with high schizotypal traits and 38 demographically matched individuals with low schizotypal traits were finally included in Study 2.

**Materials for Study 1 and Study 2**

Goals are classified into approach and avoidance goals. Approach goals refer to positive goals that the individual strives to achieve (positive events that the individual wants to achieve, to move towards or maintain a desired or positive state). For example, “it is very important for me to get into graduate school in the future”. Avoidance goals refer to negative events that the individual avoids to happen (negative events or unwanted states that the individual wants to avoid) (Dickson & MacLeod, 2004). For example, “it is important for me to avoid failing a test in the future”. During the goal generation phase, individuals were encouraged to generate as many approach and avoidance goals as possible (at least six for each). After that, they were required to select six most important approach goals and six most important avoidance goals, and then rated these goals for importance, possibility of fulfilment, centrality (the central role of the individual plays in achieving the goal), difficulty of fulfilment, sense of happiness if the goal is achieved and sense of sadness if the goal is not achieved on a 0 – 100 scale (0 = not at all, 100 = very much). For the goal-unrelated cues refer to events that are likely to occur in the future and relevant to oneself, but the content of the event is not personal goal-related (e.g., going to a new restaurant with classmates next Monday). In this study, 16 goal-unrelated cues were extract from the literature (e.g., walking, shopping). Participants need to choose 6 cue words that best fit their individual situation from the provided list. Finally, 6 approach goals, 6 avoidance goals, and 6 goal-unrelated cues were identified for each participant to be used for the second stage. Therefore, each participant had their own goal-related cues and goal-unrelated cues reflective of their unique circumstances (Jeunehomme & D'Argembeau, 2021).

**Study 1 Procedure**

The present study employed a block design, participants completed all cue words under one personal goal condition, then proceeded to another condition. The order of the three personal goal conditions was counterbalanced across participants. Participants were instructed to imagine or recall a related event based on the cue and provide a detailed description without any time limit. Following the description, participants need to complete ratings, including vividness, sense of experience, emotional valence, emotional intensity and difficulty.

Prior to the formal test, participants completed a practice session, the experimenter explained all requirements and procedures. In addition, the experimenter explained the criteria of a specific event and provided an example of a specific event. The formal test procedure is the same as practice, if a participant was unable to generate any event, the experimenter would provide a prompt ("Can you think of any event?"). If no events could be generated even after prompting, the next cue was presented. The entire process was audio recorded, and the descriptions were transcribed for subsequent coding purposes.

**Study 2 Procedure**

The procedure of Study 2 for behavioral task was consistent with Study 1. After completing the behavioral tasks, participants were required to undertake a resting-state functional MRI scanning.

**Coding of specificity for Study 1 and Study 2**

The rating criteria were adapted from the Autobiographical Memory Test manual (D'Argembeau, Raffard, & Van der Linden, 2008; Williams, Teasdale, Segal, & Soulsby, 2000; Yang et al., 2018). Events were classified into four categories:

*Specific events:* a particular event lasting no more than 24 hours, and includes specific details such as time, place, perceptual details, and thoughts, etc.;

*Extended event:* a particular event lasting more than 24 hours, and includes some details;

*Categorical event:* events that occur repeatedly or summarize a type of event;

*Omission:* did not mention any events or did not describe events as required.

The proportion of specific events was used as the index of specificity. Two trained raters rated a proportion of responses independently, and the inter-rater reliability (Cohen’s Kappa) was 0.848 (Cohen, 1960). The final scores included in the analysis were provided by one rater blinded of the group status of participants.

**Study 2 Preprocessing and denoising of MRI data**

The preprocessing pipeline has the following steps: (1) DICOM data were converted to NIfTI format, and the first five time points were removed; (2) slice-timing correction; (3) spatial realignment to the first volume and unwarping; (4) outlier detection based on the ART tool (<http://www.nitrc.org/projects/artifact_detect>). Volumes were identified as outliners if the average intensity deviated more than three standard deviations from the global mean intensity or the composite head movement exceeded 0.5 mm from the previous volume. Participants with a total number of outliners exceeds 20% of the total points (46 outlier time points in the present study) would be excluded in subsequent analysis (Nalci, Rao, & Liu, 2017); (5) co-registration of structural and functional images; (6) segmentation of the structural image into gray matter, white matter, and cerebrospinal fluid; (7) denoising using the anatomic component-based noise correction method (aCompCor), white matter and cerebrospinal fluid components, the six head motion parameters and number of abnormal time points were used as regression variables to remove the physiological noise (Behzadi, Restom, Liau, & Liu, 2007); (8) normalization in Montreal Neurological Institute (MNI) space; (9) smoothing with a 6 mm full-width at half maximum Gaussian kernel; (10) band-pass filtering of 0.01-0.1 Hz to remove high-frequency and low-frequency drift.

**Table S1.** The regions of interest related to core network and personal goal processing

| No | ROI | Hemisphere | MNI coordinates | | |
| --- | --- | --- | --- | --- | --- |
|  |  |  | X | Y | Z |
| **Core network (Fornara et al., 2017)** | | | | | |
| 1 | Middle Frontal Gyrus | L | -26 | 10 | 58 |
| 2 | Middle Frontal Gyrus | R | 0 | 56 | -2 |
| 3 | Middle Temporal Gyrus | L | -58 | -8 | -20 |
| 4 | Middle Temporal Gyrus | R | 48 | -4 | -38 |
| 5 | Hippocampus | L | -20 | -20 | -14 |
| 6 | Hippocampus | R | 22 | -26 | -10 |
| 7 | Precuneus | L | -4 | -58 | 18 |
| 8 | Precuneus | R | 6 | -56 | 50 |
| **Personal goal processing (Stawarczyk & D'Argembeau, 2015)** | | | | | |
| 9 | Middle Frontal Gyrus | L | -4 | 50 | -10 |
| 10 | Middle Frontal Gyrus | R | 4 | 50 | -10 |

Notes: MNI: Montreal Neurological Institute. L = left; R = right.

**Additional results**

**Study 1 Results**

There were no significant differences between the two groups of participants on age, length of education, estimated IQ, verbal fluency, and characteristics of goals (See Table S2 & Table S3).

**Table S2.** The basic information of participants in Study 1

|  | HST (N = 37) | | LST (N = 40) | | t/χ^2^  *_df_* _=（75）_ | *p* | Cohen's *d* |
| --- | --- | --- | --- | --- | --- | --- | --- |
|  | Mean | SD | Mean | SD |  |  |  |
| Age | 21.16 | 2.78 | 21.18 | 2.51 | -0.02 | 0.983 | -0.01 |
| Length of education (years) | 14.89 | 1.73 | 15.08 | 2.20 | -0.40 | 0.687 | -0.92 |
| Gender (female: male) | 26:11 | | 29:11 | | 0.05 | 0.829 |  |
| Estimated IQ | 118.00 | 14.00 | 121.00 | 12.93 | -0.98 | 0.331 | -0.22 |
| Verbal Fluency | 24.59 | 4.47 | 24.82 | 2.97 | 0.26 | 0.799 | 0.06 |
| SPQ | 49.30 | 7.13 | 15.88 | 6.66 | 21.26 | < 0.001 | 4.85 |
| SPQ_cognitive | 19.83 | 6.41 | 8.82 | 4.89 | 8.04 | < 0.001 | 1.97 |
| SPQ_interpersonal | 21.47 | 6.14 | 7.11 | 4.47 | 11.16 | < 0.001 | 2.73 |
| SPQ_disorganized | 10.23 | 4.31 | 2.74 | 2.37 | 9.14 | < 0.001 | 2.23 |

Note: HST = high schizotypal traits, LST = low schizotypal traits; SPQ = schizotypal personality questionnaire.

**Table S3.** The characteristics of personal goals in Study 1

|  | HST (N = 37) | | | | LST (N = 40) | | | | Group | Goal type | Group x Goal type |
| --- | --- | --- | --- | --- | --- | --- | --- | --- | --- | --- | --- |
|  | Approach | | Avoidance | | Approach | | Avoidance | | F  *_df_* _=（1,75）_ | F  *_df_* _=（1,75）_ | F  *_df_* _=（1,75）_ |
|  | Mean | SD | Mean | SD | Mean | SD | Mean | SD |  |  |  |
| Importance | 85.80 | 7.92 | 85.97 | 7.83 | 85.72 | 9.12 | 86.61 | 8.96 | 0.03 | 0.48 | 0.21 |
| Possibility | 69.43 | 8.71 | 71.43 | 9.37 | 73.68 | 9.96 | 72.93 | 10.12 | 2.33 | 0.32 | 1.54 |
| Centrality | 63.58 | 12.18 | 66.08 | 13.73 | 67.12 | 10.88 | 67.63 | 9.94 | 1.14 | 1.60 | 0.70 |
| Difficulty | 72.49 | 12.19 | 68.41 | 12.42 | 68.51 | 12.41 | 63.59 | 13.92 | 3.00 | 9.97^**^ | 0.09 |
| Sense of happiness | 84.95 | 10.86 | 77.78 | 14.46 | 86.60 | 10.42 | 79.11 | 14.38 | 0.32 | 35.59^***^ | 0.02 |
| Sense of sadness | 70.55 | 14.96 | 72.59 | 16.04 | 65.99 | 13.67 | 72.79 | 10.98 | 0.61 | 7.86^**^ | 2.31 |

Note: ** *p* < 0.01; *** *p* < 0.001; HST = high schizotypal traits, LST = low schizotypal traits. Group = high schizotypal traits vs low schizotypal traits; Goal type = Approach vs Avoidance; Group x Goal type = Group and Goal type interaction.

2 (Group: HST, LST) * 3 (Goal type: Approach personal goals, Avoidance personal goals, Personal goal-unrelated) * 2 (Time orientation: AM, EFT) repeated measure ANOVAs were used to analyze MTT indexes (Table S4). There was a significant interaction between Group and Goal type in specificity (F _(2,150)_ = 3.97, *p* = 0.021, η_p_² = 0.05). Simple effect analysis revealed that the LST generated more specific events with both approach goals (t _(75)_ = 4.05, *p* < 0.001, Cohen’s *d* = 0.56) and avoidance goals (t _(75)_ = 4.30, *p* = 0.001, Cohen’s *d* = 0.59) than personal goal-unrelated cues, whereas HST did not show significant differences among three types of goals (Approach goals: t _(75)_ = 1.68, *p* = 0.248, Cohen’s *d* = 0.24) and avoidance goals (t _(75)_ = 0.23, *p* =0.999, Cohen’s *d* = 0.03). These results suggest that HST exhibited reduced “personal goal-advantage effect” in specificity.

The main effect of Group was significant in specificity (F _(1,75)_ = 6.26, *p =* 0.014, η_p_²*=* 0.08) and emotional valence (F _(1, 75)_ = 6.55, *p =* 0.012, η_p_² = 0.08). HST generated fewer specific events and with less positive emotion compared to LST.

The main effect of Goal type was significant in specificity (F _(2,150)_ = 8.93, *p* < 0.001, η_p_² = 0.11), vividness (F _(2,150)_ = 18.41, *p* < 0.001, η_p_² = 0.20), sense of experience (F _(2,150)_ = 32.29, *p* < 0.001, η_p_² = 0.30), emotional valence (F _(2,150)_ = 55.51, *p* < 0.001, η_p_² = 0.43), emotional intensity (F _(2,150)_ = 33.78, *p* < 0.001, η_p_²= 0.31), and difficulty (F _(2,150)_ = 4.81, *p* = 0.009, η_p_² = 0.06). Simple effect analysis revealed that approach goals related events showed higher specificity (t _(75)_ = 4.02, *p* < 0.001, Cohen’s *d* = 0.40), vividness (t _(75)_ = 5.43, *p* < 0.001, Cohen’s *d* = 0.62), sense of experience (t _(75)_ = 7.03, *p* < 0.001, Cohen’s *d* = 0.80), emotional valence (t _(75)_ = 5.22, *p* < 0.001, Cohen’s *d* = 0.60), emotional intensity (t _(75)_ = 7.55, *p* < 0.001, Cohen’s *d* = 0.86) and less difficulty (t _(75)_ = -2.74, *p* = 0.023, Cohen’s *d* = -0.31) than personal goal-unrelated events. Avoidance goals related events showed higher specificity (t _(75)_ = 3.15, *p* = 0.015, Cohen’s *d* = 0.31), vividness (t _(75)_ = 5.05, *p* < 0.001, Cohen’s *d* = 0.58), sense of experience (t _(75)_ = 6.00, *p* < 0.001, Cohen’s *d* = 0.68), less positive emotional valence (t _(75)_ = -5.48, *p* < 0.001, Cohen’s *d* = -0.62) and higher emotional intensity (t _(75)_ = 5.36, *p* < 0.001, Cohen’s *d* = 0.61) than personal goal-unrelated events. Approach goals related events showed more positive emotional valence (t _(75)_ = 10.23, *p* < 0.001, Cohen’s *d* = 1.17) and higher emotional intensity (t _(75)_ = 2.46, *p* = 0.049, Cohen’s *d* = 0. 28) than avoidance goals.

**Table S4.** The phenomenological characteristics of personal goal related and personal goal-unrelated MTT in Study 1

|  |  | HST | | | | | | LST | | | | | | Group | | Goal  type | | | Group x Goal type | | | |  |
| --- | --- | --- | --- | --- | --- | --- | --- | --- | --- | --- | --- | --- | --- | --- | --- | --- | --- | --- | --- | --- | --- | --- | --- |
|  |  | Approach | | Avoidance | | Non-personal | | Approach | | Avoidance | | Non-personal | | F | η_p_² | | F | η_p_² | | F | η_p_² |  |  |
|  |  | Mean | SD | Mean | SD | Mean | SD | Mean | SD | Mean | SD | Mean | SD | *_df_* _=（1,75）_ |  | | *_df_* _=（2,150）_ |  | | *_df_* _=（2,150）_ |  | | |
| Specificity^#^ | AM | 0.69 | 0.24 | 0.62 | 0.27 | 0.64 | 0.26 | 0.74 | 0.25 | 0.76 | 0.24 | 0.68 | 0.25 | 6.26^*^ | 0.08 | | 8.93^***^ | 0.11 | | 3.97^*^ | 0.05 | | |
| (0-1) | EFT | 0.65 | 0.25 | 0.62 | 0.24 | 0.58 | 0.21 | 0.79 | 0.18 | 0.78 | 0.21 | 0.58 | 0.29 |  |  | |  |  | |  |  | | |
| Vividness | AM | 7.36 | 1.32 | 7.27 | 1.15 | 7.10 | 1.05 | 7.55 | 1.23 | 7.67 | 1.06 | 7.32 | 1.14 | 1.86 | 0.02 | | 18.41^***^ | 0.20 | | 1.16 | 0.02 | | |
| (1-10) | EFT | 6.85 | 1.31 | 6.59 | 1.34 | 6.01 | 1.20 | 7.03 | 1.28 | 7.11 | 1.27 | 6.40 | 1.21 |  |  | |  |  | |  |  | | |
| Sense of experience | AM | 7.36 | 1.51 | 7.46 | 1.48 | 7.03 | 1.27 | 7.64 | 1.15 | 7.74 | 0.84 | 7.17 | 1.06 | 1.49 | 0.02 | | 32.29^***^ | 0.30 | | 0.85 | 0.01 | | |
| (1-10) | EFT | 6.50 | 1.37 | 6.04 | 1.39 | 5.29 | 1.40 | 6.54 | 1.30 | 6.60 | 1.38 | 5.67 | 1.28 |  |  | |  |  | |  |  | | |
| Valence | AM | 3.15 | 0.57 | 2.75 | 0.64 | 3.17 | 0.33 | 3.30 | 0.45 | 2.84 | 0.47 | 3.21 | 0.35 | 6.55^*^ | 0.08 | | 55.51^***^ | 0.43 | | 0.21 | <0.01 | | |
| (1-5) | EFT | 3.65 | 0.66 | 2.85 | 0.67 | 3.21 | 0.42 | 3.93 | 0.52 | 3.15 | 0.65 | 3.45 | 0.47 |  |  | |  |  | |  |  | | |
| Intensity | AM | 3.13 | 0.61 | 3.18 | 0.58 | 2.87 | 0.52 | 3.21 | 0.57 | 3.20 | 0.53 | 2.95 | 0.58 | 0.62 | 0.01 | | 33.78^***^ | 0.31 | | 0.70 | 0.01 | | |
| (1-5) | EFT | 3.40 | 0.67 | 3.18 | 0.64 | 2.71 | 0.48 | 3.47 | 0.60 | 3.20 | 0.56 | 2.95 | 0.68 |  |  | |  |  | |  |  | | |
| Difficulty | AM | 1.65 | 0.56 | 1.63 | 0.51 | 1.73 | 0.54 | 1.55 | 0.48 | 1.49 | 0.45 | 1.55 | 0.49 | 2.61 | 0.03 | | 4.81^**^ | 0.06 | | 0.28 | <0.01 | | |
| (1-5) | EFT | 1.93 | 0.56 | 2.07 | 0.62 | 2.21 | 0.59 | 1.78 | 0.60 | 1.85 | 0.58 | 2.00 | 0.61 |  |  | |  |  | |  |  | | |

Note: ^#^ Proportion of specific events; * *p* < 0.05; ** *p* < 0.01; *** *p* < 0.001; HST = high schizotypal traits, LST = low schizotypal traits; AM = Autobiographical memory; EFT = Episodic future thinking.

Group = high schizotypal traits vs low schizotypal traits; Time = autobiographical memory vs episodic future thinking; Goal type = Approach goals vs Avoidance goals vs Personal goal-unrelated; Group x Goal type = Group and Goal type interaction

**Study 2 Results**

There were no significant differences between the two groups of participants on age, length of education, estimated IQ, verbal fluency, and characteristics of goals (See Table S5 & Table S6).

**Table S5.** The basic information of participants in Study 2

|  | HST (N = 39) | | LST (N = 38) | | t/χ^2^  *_df_* _=（75）_ | *p* | Cohen's *d* |
| --- | --- | --- | --- | --- | --- | --- | --- |
|  | Mean | SD | Mean | SD |  |  |  |
| Age | 20.67 | 2.13 | 20.37 | 1.44 | 0.72 | 0.475 | 0.16 |
| Gender (female: male) | 20 : 19 | | 21 : 17 | | 0.12 | 0.726 |  |
| Length of education (years) | 14.46 | 1.67 | 14.55 | 1.20 | -0.27 | 0.785 | -0.06 |
| Estimated IQ | 122.64 | 10.05 | 122.34 | 9.62 | 0.13 | 0.894 | 0.03 |
| Verbal Fluency | 24.56 | 3.28 | 25.53 | 4.67 | -1.05 | 0.297 | -0.24 |
| SPQ | 48.28 | 6.78 | 16.61 | 7.56 | 19.36 | < 0.001 | 4.41 |
| SPQ_cognitive | 20.69 | 4.64 | 7.68 | 4.33 | 12.72 | < 0.001 | 2.90 |
| SPQ_interpersonal | 22.03 | 4.35 | 6.53 | 3.91 | 16.43 | < 0.001 | 3.75 |
| SPQ_disorganized | 11.23 | 2.72 | 3.76 | 3.04 | 11.36 | < 0.001 | 2.59 |

Note: HST = high schizotypal traits, LST = low schizotypal traits; SPQ = schizotypal personality questionnaire.

**Table S6.** The characteristics of personal goals in Study 2

|  | HST (N = 39) | | | | LST (N = 38) | | | | Group | Goal type | Interaction |
| --- | --- | --- | --- | --- | --- | --- | --- | --- | --- | --- | --- |
|  | Approach | | Avoidance | | Approach | | Avoidance | | F  *_df_* _=（1,75）_ | F  *_df_* _=（1,75）_ | F  *_df_* _=（1,75）_ |
|  | Mean | SD | Mean | SD | Mean | SD | Mean | SD |  |  |  |
| Importance | 83.83 | 9.98 | 86.67 | 10.19 | 83.57 | 8.50 | 82.05 | 12.04 | 1.61 | 0.25 | 2.68 |
| Possibility | 70.24 | 10.01 | 71.52 | 12.85 | 72.33 | 7.53 | 72.41 | 10.10 | 0.53 | 0.35 | 0.28 |
| Centrality | 63.33 | 13.83 | 64.26 | 14.49 | 63.20 | 9.16 | 66.18 | 12.59 | 0.12 | 2.19 | 0.60 |
| Difficulty | 71.37 | 14.99 | 64.31 | 15.67 | 70.40 | 11.00 | 60.38 | 16.41 | 0.67 | 33.06*** | 1.00 |
| Sense of happiness | 84.87 | 12.93 | 76.06 | 16.26 | 85.76 | 10.71 | 75.74 | 19.18 | 0.01 | 41.68*** | 0.17 |
| Sense of sadness | 64.71 | 15.75 | 70.12 | 18.66 | 61.62 | 16.02 | 67.46 | 16.08 | 0.82 | 7.27 | 0.01 |

Note: *** *p* < 0.001; HST = high schizotypal traits, LST = low schizotypal traits. Group = high schizotypal traits vs low schizotypal traits; Goal type = Approach vs Avoidance; Interaction = Group * Goal type.

2 (Group: HST, LST) * 3 (Goal type: Approach personal goals, Avoidance personal goals, Personal goal-unrelated) * 2 (Time: AM, EFT) repeated measure ANOVAs were used to analyze MTT indexes (See Table S7). There was a significant interaction between Group and Goal type in specificity (F _(1,2,150)_ = 3.85, *p* = 0.023, η_p_² = 0.05). Simple effect analysis revealed that the LST generated more specific events with both approach goals (t _(75)_ = 3.90, *p* < 0.001, Cohen’s *d* = 0.75) and avoidance goals (t _(150)_ = 4.26, *p* < 0.001, Cohen’s *d* = 0.82) than HST, whereas the two groups did not show significant difference on personal goal-unrelated events (t _(75)_ = 1.54, *p* = 0.102, Cohen’s *d* = 0.30).

The main effect of Group was significant in specificity (F _(1,75)_ = 16.78, *p* < 0.001, η_p_²*=* 0.18) and vividness (F _(1,75)_ = 4.19, *p* = 0.044, η_p_² = 0.05). HST generated fewer specific events and with less vividness compared to LST.

The main effect of Goal type was significant in vividness (F _(2,150)_ = 10.51, *p* < 0.001, η_p_² = 0.12), sense of experience (F _(2,150)_ = 16.56, *p* < 0.001, η_p_² = 0.18), emotional valence (F _(2,150)_ = 55.13, *p* < 0.001, η_p_² = 0.42), emotional intensity (F _(2,150)_ = 29.35, *p* < 0.001, η_p_²= 0.28), and difficulty (F _(2,150)_ = 4.43, *p* = 0.014, η_p_² = 0.06). Simple effect analysis revealed that approach goals related events showed more vividness (t _(75)_ = 3.45, *p* = 0.006, Cohen’s *d* = 0.27), sense of experience (t _(75)_ = 4.86, *p* < 0.001, Cohen’s *d* = 0.35), higher emotional valence (t _(75)_ = 4.12, *p* < 0.001, Cohen’s *d* = 0.47) and higher emotional intensity (t _(75)_ = 7.35, *p* < 0.001, Cohen’s *d* = 0.65) than personal goal-unrelated events. Avoidance goals related events showed higher vividness (t _(75)_ = 4.34, *p* < 0.001, Cohen’s *d* = 0.34), sense of experience (t _(75)_ = 5.10, *p* < 0.001, Cohen’s *d* = 0.37), and less positive emotional valence (t _(75)_ = -6.31, *p* < 0.001, Cohen’s *d* = -0.72) and higher emotional intensity (t _(75)_ = 5.55, *p* < 0.001, Cohen’s *d* = 0.49) than personal goal-unrelated events. Approach goals related events showed more vividness (t _(75)_ = 4.34, *p* < 0.001, Cohen’s *d* = 0.34), higher emotional valence (t _(75)_ = 10.42, *p* < 0.001, Cohen’s *d* = 1.19) than avoidance goals.

***Associations between resting-state Functional Connectivity and “personal goal-advantage effect”***

The pattern of association between resting-state functional connectivity and the “personal goal-advantage effect” in MTT showed differences between HST and LST (See Figure S1 & Table S8).

As shown in Figure S1 Panel A, the correlation between the functional connectivity of the right middle temporal gyrus (MTG_R) and the right supramarginal gyrus and vividness showed significant group difference (t = 5.07, *p*_FWE_ = 0.025). The association was positive in LST (*r* = 0.56, *p* < 0.001), but negative in HST (*r* = -0.49, *p* = 0.009).

As shown in Figure S1 Panel B, the correlation between the functional connectivity of the left hippocampus (HIP_L) and the left insula and sense of experience showed significant group difference (t = 5.80, *p*_FWE_ = 0.023). The association was positive in LST (*r* = 0.53, *p* = 0.001), but negative in HST (*r* = -0.48, *p* = 0.003).

As shown in Figure S1 Panel C, the correlation between the functional connectivity of the left middle frontal gyrus (MFG_L) and the left frontal pole and emotional valence showed significant group difference (t = -5.52, *p*_FWE_ = 0.017). There was no relationship in LST (*r* = -0.01, *p* = 0.990), but positive in HST (*r* = 0.61, *p* < 0.001).

As Figure S1 Panel D, the correlation between the functional connectivity of the right precuneus (Precuneus_R) and the left postcentral gyrus and emotional valence showed significant group difference (t = -5.20, *p*_FWE_ = 0.001). There was no relationship in LST (*r* = -0.04, *p* = 0.807), but positive in HST (*r* = 0.67, *p* < 0.001).

As Figure S1 Panel E, the correlation between the functional connectivity of the left hippocampaus (HIP_L) and the right temporal fusiform gyrus and emotional intensity showed significant group difference (t = -7.47, *p*_FWE_ = 0.001). The association was negative in LST (*r =* -0.58, *p* < 0.001), but positive in HST (*r* = 0.65, *p* < 0.001).

As Figure S1 Panel F, the correlation between the functional connectivity of the left middle frontal gyrus and the right supramarginal gyrus and emotional intensity showed significant group difference (t = 4.93, *p*_FWE_ = 0.032,). There was no relationship in LST (*r* = 0.28, *p* = 0.101), but negative in HST (*r* = -0.54, *p* < 0.001).

After multiple comparison correction (*p*_FWEcorrection_ = 0.05/10ROIs), the significant group differences in correlations between the right precuneus and the left postcentral gyrus and emotional valence (Figure S1 Panel D), and the left hippocampus and the right temporal fusiform gyrus and emotional intensity (Figure S1 Panel E) remained significant, these results were reported in the main text.

Table S7. The group difference of the MTT phenomenological characteristics in each type of personal goal in Study 2

|  |  | HST | | | | | | LST | | | | | | Group | | Goal  type | | Group x Goal type | |
| --- | --- | --- | --- | --- | --- | --- | --- | --- | --- | --- | --- | --- | --- | --- | --- | --- | --- | --- | --- |
|  |  | Approach | | Avoidance | | Non-personal | | Approach | | Avoidance | | Non-personal | | F | η_p_² | F | η_p_² | F | η_p_² |
|  |  | Mean | SD | Mean | SD | Mean | SD | Mean | SD | Mean |  | Mean | SD | *_df_* _=（1,75）_ |  | *_df_* _=（2,150）_ |  | *_df_* _=（2,150）_ |  |
| Specificity^#^ | AM | 0.60 | 0.27 | 0.52 | 0.28 | 0.64 | 0.26 | 0.68 | 0.24 | 0.73 | 0.24 | 0.70 | 0.22 | 16.78^***^ | 0.18 | 1.20 | 0.02 | 3.85* | 0.05 |
| (0-1) | EFT | 0.45 | 0.29 | 0.54 | 0.25 | 0.60 | 0.28 | 0.75 | 0.24 | 0.75 | 0.25 | 0.70 | 0.24 |  |  |  |  |  |  |
| Vividness | AM | 7.24 | 1.56 | 7.08 | 1.28 | 7.01 | 1.42 | 7.51 | 1.21 | 7.76 | 1.16 | 7.36 | 1.26 | 4.19^*^ | 0.05 | 10.51^***^ | 0.12 | 0.35 | 0.01 |
| (1-10) | EFT | 6.23 | 1.33 | 6.45 | 1.26 | 5.82 | 1.41 | 6.93 | 1.20 | 6.96 | 1.17 | 6.33 | 1.27 |  |  |  |  |  |  |
| Sense of experience | AM | 7.19 | 1.55 | 7.06 | 1.38 | 6.88 | 1.53 | 7.42 | 1.30 | 7.51 | 1.19 | 7.00 | 1.32 | 1.18 | 0.02 | 16.56^***^ | 0.18 | 0.38 | 0.01 |
| (1-10) | EFT | 5.88 | 1.49 | 6.03 | 1.20 | 5.33 | 1.78 | 6.31 | 1.33 | 6.29 | 1.42 | 5.60 | 1.45 |  |  |  |  |  |  |
| Valence | AM | 3.25 | 0.46 | 2.70 | 0.57 | 3.16 | 0.45 | 3.32 | 0.56 | 2.79 | 0.53 | 3.32 | 0.30 | 2.33 | 0.03 | 55.13^***^ | 0.42 | 0.89 | 0.01 |
| (1-5) | EFT | 3.76 | 0.59 | 3.13 | 0.62 | 3.28 | 0.42 | 3.91 | 0.53 | 3.08 | 0.77 | 3.47 | 0.46 |  |  |  |  |  |  |
| Intensity | AM | 3.14 | 0.54 | 3.03 | 0.52 | 2.88 | 0.55 | 3.13 | 0.67 | 3.16 | 0.65 | 2.82 | 0.78 | 0.01 | <0.01 | 29.35^***^ | 0.28 | 1.04 | 0.01 |
| (1-5) | EFT | 3.28 | 0.67 | 3.17 | 0.59 | 2.83 | 0.60 | 3.38 | 0.63 | 3.15 | 0.63 | 2.71 | 0.86 |  |  |  |  |  |  |
| Difficulty | AM | 1.62 | 0.61 | 1.59 | 0.52 | 1.64 | 0.46 | 1.48 | 0.40 | 1.48 | 0.47 | 1.54 | 0.55 | 1.57 | 0.02 | 4.43^*^ | 0.06 | 0.38 | 0.01 |
| (1-5) | EFT | 1.99 | 0.55 | 1.91 | 0.56 | 2.20 | 0.73 | 1.81 | 0.61 | 1.86 | 0.54 | 2.01 | 0.68 |  |  |  |  |  |  |

Note: ^#^ Proportion of specific events; * *p* < 0.05; *** *p* < 0.001; HST = high schizotypal traits, LST = low schizotypal traits; AM = Autobiographical memory; EFT = Episodic future thinking. Groups = high schizotypal traits vs low schizotypal traits; Goal type = Approach goals vs Avoidance goals vs Personal goal-unrelated, Interaction = Groups * Goal type.


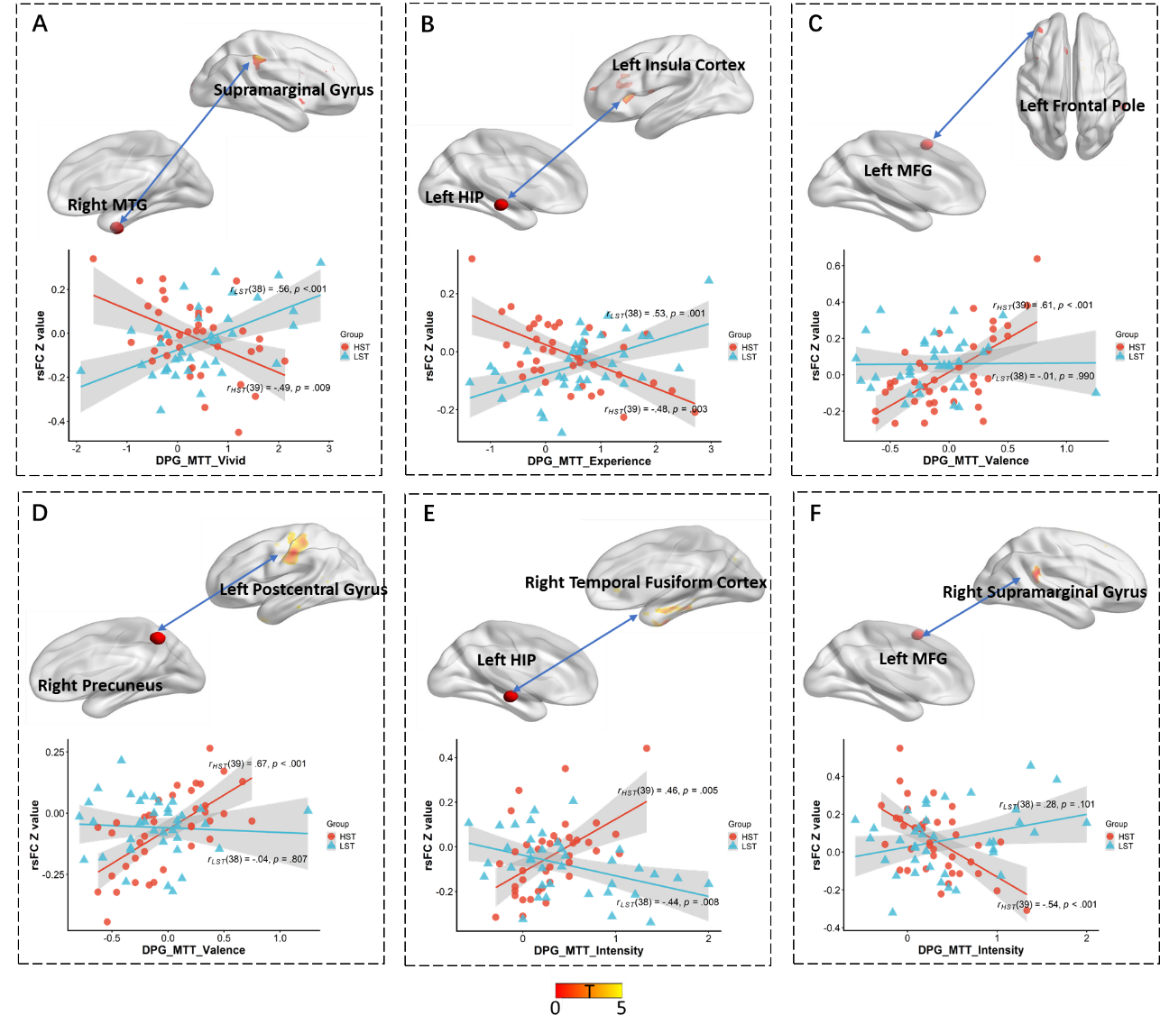


**Figure S1.** Significant group difference of the relationship between personal goal-advantage effect on MTT and functional connectivity

Note: MTT= mental time travel; L = left; R = right. Panel A shows the positive correlation between the functional connectivity of the right middle temporal gyrus (MTG_R) and the right supramarginal gyrus with personal goal-advantage effect on vividness in LST, but this correlation was negative in HST. Panel B shows the positive correlation between the functional connectivity of the left hippocampal (HIP_L) and the left insula with personal goal-advantage effect on sense of experience in LST, but this correlation was negative in HST. Panel C shows non-significant correlation between the functional connectivity of the left middle frontal gyrus (MFG_L) and the left frontal pole with personal goal-advantage effect on emotional valence in LST, but this correlation was positive in HST. Panel D shows non-significant correlation between the functional connectivity of the right precuneus (Precuneus_R) and the left postcentral gyrus with personal goal-advantage effect on emotional valence in LST, but this correlation was positive in HST. Panel E shows the negative correlation between the functional connectivity of the left hippocampus and the right temporal fusiform gyrus with personal goal-advantage effect on emotional intensity in LST, but this correlation was positive in HST. Panel F shows non-significant relationship correlation between the functional connectivity of the left middle frontal gyrus and the right supramarginal gyrus with personal goal-advantage effect on emotional intensity in LST, but this correlation was negative in HST. Threshold: cluster-level FWE adjusted *p* < 0.005, cluster size > 20 (Voxel-level uncorrected *p* < 0.005).

**Table S8.** The group difference of the relationship between the personal goal-advantage effect in MTT and functional connectivity

| Personal Goal-Advantage Effect | ROIs | Regions | T | MNI coordinates | | | Cluster size | *p*_FWE_ | HST  (*r , p*) | LST  (*r , p*) |
| --- | --- | --- | --- | --- | --- | --- | --- | --- | --- | --- |
|  |  |  |  | X | Y | Z |  |  |  |  |
| Specificity^#^ | NAN |  |  |  |  |  |  |  |  |  |
| Vividness | Right Middle Temporal Gyrus | Right Supramarginal Gyrus | 5.07 | 54 | -33 | 42 | 48 | 0.025 | -0.49  0.009 | 0.56  < 0.001 |
| Sense of experience | Left Hippocampus | Left Insular Cortex | 5.80 | -27 | 21 | 12 | 47 | 0.023 | -0.48  0.003 | 0.53  0.001 |
| Valence | Left Middle Frontal Gyrus | Left Frontal Pole | -5.52 | -27 | 48 | 33 | 52 | 0.017 | 0.61  < 0.001 | -0.01  0.990 |
|  | Right Precuneus | Left Postcentral Gyrus | -5.20 | -54 | -21 | 42 | 70 | 0.001 | 0.67  < 0.001 | -0.04  0.807 |
| Intensity | Left Hippocampus | Right Temporal Fusiform Cortex | -7.47 | 42 | -6 | -39 | 76 | 0.001 | 0.65  < 0.001 | -0.58  < 0.001 |
|  | Left Middle Frontal Gyrus | Right Supramarginal Gyrus | 4.93 | 69 | -36 | -12 | 45 | 0.032 | -0.54  < 0.001 | 0.28  0.101 |
| Difficulty | NAN |  |  |  |  |  |  |  |  |  |

Note: MTT= mental time travel; HST = high schizotypal traits; LST = low schizotypal traits; Threshold: cluster-level FWE corrected *p* < 0.005, cluster size > 20 (Voxel-level uncorrected *p* < 0.005).

**References:**

Behzadi, Y., Restom, K., Liau, J., & Liu, T. T. (2007). A component based noise correction method (CompCor) for BOLD and perfusion based fMRI. *NeuroImage, 37*(1), 90-101. doi:10.1016/j.neuroimage.2007.04.042

Chen, W. J., Hsiao, C. K., & Lin, C. C. H. (1997). Schizotypy in community samples: The three-factor structure and correlation with sustained attention. *Journal of Abnormal Psychology, 106*(4), 649-654. doi:10.1037/0021-843X.106.4.649

Cohen, J. (1960). A Coefficient of Agreement for Nominal Scales. *Educational and Psychological Measurement, 20*(1), 37-46. doi:10.1177/001316446002000104

D'Argembeau, A., Raffard, S., & Van der Linden, M. (2008). Remembering the past and imagining the future in schizophrenia. *Journal of Abnormal Psychology, 117*(1), 247-251. doi:10.1037/0021-843x.117.1.247

Dickson, J. M., & MacLeod, A. K. (2004). Approach and Avoidance Goals and Plans: Their Relationship to Anxiety and Depression. *Cognitive Therapy and Research, 28*(3), 415-432. doi:10.1023/B:COTR.0000031809.20488.ee

Faul, F., Erdfelder, E., Lang, A. G., & Buchner, A. (2007). G*Power 3: a flexible statistical power analysis program for the social, behavioral, and biomedical sciences. *Behavior Research Methods, 39*(2), 175-191. doi:10.3758/bf03193146

Jeunehomme, O., & D'Argembeau, A. (2021). The role of self-reference and personal goals in the formation of memories of the future. *Memory and Cognition, 49*(6), 1119-1135. doi:10.3758/s13421-021-01150-9

Nalci, A., Rao, B. D., & Liu, T. T. (2017). Global signal regression acts as a temporal downweighting process in resting-state fMRI. *NeuroImage, 152*, 602-618. doi:10.1016/j.neuroimage.2017.01.015

Raine, A. (1991). The SPQ: A Scale for the Assessment of Schizotypal Personality Based on DSM-III-R Criteria. *Schizophrenia Bulletin, 17*(4), 555-564. doi:10.1093/schbul/17.4.555

Sheehan, D. V., Lecrubier, Y., Sheehan, K. H., Amorim, P., Janavs, J., Weiller, E., . . . Dunbar, G. C. (1998). The Mini-International Neuropsychiatric Interview (M.I.N.I.): the development and validation of a structured diagnostic psychiatric interview for DSM-IV and ICD-10. *Journal of Clinical Psychiatry, 59 Suppl 20*, 22-33;quiz 34-57.

Si, T. M., Shu, L., Dang, W. M., Se, Y. A., Chen, J. X., Dong, W. T., . . . Zhang, W. H. (2009). Evaluation of the reliability and validity of Chinese version of the Mini-International Neuropsychiatric Interview in patients with mental disorders. *Chinese Mental Health Journal, 23*(7), 493-503.

Williams, J. M. G., Teasdale, J. D., Segal, Z. V., & Soulsby, J. (2000). Mindfulness-based cognitive therapy reduces overgeneral autobiographical memory in formerly depressed patients. *Journal of Abnormal Psychology, 109*(1), 150-155. doi:10.1037/0021-843x.109.1.150

Yang, Z. Y., Xie, D. J., Zou, Y. M., Wang, Y., Li, Y., Shi, H. S., . . . Chan, R. C. K. (2018). Prospection deficits in schizophrenia: Evidence from clinical and subclinical samples. *Journal of Abnormal Psychology, 127*(7), 710-721. doi:10.1037/abn0000382
